# A Case of Probable Amisulpride Induced Mania after Eight Months of Therapy

**DOI:** 10.1155/2017/6976917

**Published:** 2017-10-09

**Authors:** Prakash Thapa, Rajasee Sharma

**Affiliations:** ^1^Department of Psychiatry, Manipal Teaching Hospital, Fulbari 11, Pokhara, Nepal; ^2^Manipal Teaching Hospital, Fulbari 11, Pokhara, Nepal

## Abstract

Development of manic symptoms during treatment with atypical antipsychotics can be a troublesome side effect that has been described with most atypical antipsychotics. However, reports of amisulpride induced mania have been rare. Here, we report the case of an 18-year-old male patient diagnosed with schizophrenia, who developed manic symptoms while on treatment with amisulpride. While previous reports have described occurrence of mania within days to three months of treatment with amisulpride, we report a case where manic symptoms occurred after around eight months of therapy. We have also attempted to describe the possible risk factors based on the available case studies.

## 1. Introduction

Emergence of manic symptoms during therapy with atypical antipsychotics has been described with most atypical antipsychotics [1]. However, manic/hypomanic symptoms occurring during therapy with amisulpride have been infrequently reported. PubMed search using the keywords “amisulpride induced mania” and “sultopride induced mania” identified only four previous relevant records [2–5]. Here, we describe a probable case of amisulpride induced mania in a patient who was diagnosed with schizophrenia. Furthermore, we have attempted to identify common risk factors in the reported cases.

## 2. Case Report

An 18-year-old male presented with symptoms of social withdrawal, academic decline, and negligence of self-care, which had started around a year back. Around one week before presentation to the hospital, the patient developed florid psychotic features like hearing voices, suspiciousness towards neighbors, locking himself up in his room as he used to say that he was fearful that people are trying to harm him, refusal of food due to suspiciousness, and disturbed biorhythm. When the patient was seen in the hospital, the patient appeared unkempt as well as agitated. On mental state examination, he had 3rd-person auditory hallucination where the patient used to hear voices discussing amongst themselves the patient, bizarre delusion, and delusion of reference and persecution. The patient was started on risperidone 4 mg, which was gradually increased to 10 mg together with trihexyphenidyl 4 mg. After 3 months of medication intake and gradual improvement in symptoms, the patient discontinued medication on his own.

On follow-up, risperidone was restarted, but as the patient was noncompliant to the medication for the second time, he was prescribed depot fluphenazine. He was managed on this for the next 3 months, during which he showed some improvement. Then, due to the lack of availability of depot fluphenazine, the patient was started on amisulpride 500 mg. He was managed on the same dose for around 6 months during which time he showed improvement. His positive symptoms gradually subsided; however, he had persistence in negative symptoms in the form of lack of initiation and social withdrawal. The medication was then decreased to 300 mg due to persistence of negative symptoms. After around 3 months of maintenance at 300 mg, the patient developed manic features in the form of elated mood, overactivity, overgrooming, overtalkativeness, and decreased need for sleep. Young's mania rating scale (YMRS) score was 34 when he was evaluated after one week of the onset of symptoms. He had no delusions or hallucinations during this time and there was no history of illicit substance use. Amisulpride was then stopped and the patient was managed on olanzapine (20 mg), lithium (900 mg), and clonazepam (1 mg). When the patient was evaluated for the second time during follow-up after 10 days, his manic symptoms had improved and his YMRS score was 14. His symptoms then gradually subsided over the next 5-6 weeks.

## 3. Discussion

 Here, we reported a case of a patient with schizophrenia, who developed florid manic symptoms while on treatment with amisulpride. Though there have been previous reports of atypical antipsychotic induced mania or hypomania, amisulpride induced mania has been reported quite rarely. We applied the Naranjo Adverse Drug Reaction Probability Scale, which yielded the score of 7, indicating probable drug reaction [6].

The first case of amisulpride induced mania was described by Murphy in 2003, in a 17-year-old girl with a history of schizophrenia [2]. Development of manic symptoms was described after 3 months of treatment with amisulpride 400 mg. However, the patient was also taking citalopram 20 mg/day, which might have contributed to the development of symptoms. The second case describes a 14-year-old male patient with interictal psychosis of 3-month duration [3]. Manic symptoms were noted in this case after 3 months of treatment with amisulpride 200 mg together with oxcarbazepine.

Another case report from India describes an 18-year-old male, who was under treatment for schizophrenia with risperidone 4 mg. Manic symptoms developed in this case within 2 weeks of adding amisulpride 50–100 mg/day. The symptoms subsided within 2 weeks when amisulpride was withdrawn and lorazepam 4 mg was initiated [4].

Amisulpride induced mania has also been described in a 19-year-old Taiwanese male, with a history of schizophrenia and cerebral palsy [5]. In this case, rapid onset of manic symptoms was noted after initiation of amisulpride. Similarly, rapid resolution of manic symptoms was achieved after switching amisulpride with risperidone. Apart from the first case described by Murphy, all patients with amisulpride induced mania were young males. A comorbid medical condition has been described in 2 cases, that is, cerebral palsy and epilepsy. Most of the cases described the onset of manic symptoms, with lower doses of amisulpride at 50–400 mg/day. Manic symptoms in all cases resolved after discontinuation of amisulpride and switching to another antipsychotic or benzodiazepine. Our report also describes the onset of symptoms of mania in a young male diagnosed with schizophrenia on amisulpride 300 mg. However, our case is different from previous reports, as the development of manic symptoms occurred after around 8 months of treatment with amisulpride.

Amisulpride has a high affinity for presynaptic D2 and D3 receptor subtypes at lower doses, with preference for limbic dopamine receptor together with affinity for dopamine autoreceptors, whereas at high doses it blocks the postsynaptic D2 receptors. At lower doses, due to the action on the presynaptic autoreceptors, there is increased dopaminergic transmission. So, at low doses, amisulpride leads to an increase in dopaminergic activity, which could explain the onset of manic symptoms [7].

We acknowledge the following limitations of our case study. Firstly, we could not obtain the blood level of amisulpride during the manic attack. Secondly, we started antimanic therapy promptly as the manic symptoms were severe. Resolution of symptoms with just discontinuation of amisulpride would have provided further support for the causal nature of association between the drug and manic symptoms. Finally, we have not rechallenged the patient with amisulpride to provoke manic symptoms due to ethical reasons.

In conclusion, we have reported a probable case of amisulpride induced mania in an 18-year-old male patient diagnosed with schizophrenia. Our case suggests that it may be prudent to be watchful for occurrence of manic symptoms in young patients with schizophrenia even if the patient has been stable on amisulpride for quite some time.
